# Hepatitis A Virus: Seroepidemiological Study in Fars Province

**DOI:** 10.5812/kowsar.1735143X.714

**Published:** 2011-08-01

**Authors:** Gholam Ali Ghorbani

**Affiliations:** 1 Baqiyatallah Research Center for Gastroenterology and Liver Diseases, Baqiyatallah University of Medical Sciences, Tehran, IR Iran

**Keywords:** Hepatitis A virus, Epidemiology, Iran

Dear Editor,

I read the article on "Seroprevalence study of hepatitis A virus in Fars province, southern Iran" that published in one of previous issues of Hepatitis Monthly by Taghavi SA et al. in which authors recommended hepatitis A vaccination in five year old children [1]. In developed countries the first dose of hepatitis A vaccination injects in one year old children while some other researchers recommend second dose after 9-15 months. There are Two main methods to prevent hepatitis A infection: 1) passive immunoprophylaxis; 2) active immunoprophylaxis through vaccination [2]. In developing countries that almost all adults carry HAV antibody, general vaccination isn't cost benefit but due to reduction in HAV infection in children, it seems that vaccination may be requirement, on the other hand, prevalence of HAV differs in developed and developing countries, therefore, the best age for vaccination against HAV may be different.

Iran is an endemic area for hepatitis A infection but the prevalence varies in different provinces [3]. Recently, improvement on both environment sanitation and healthy water and food has decreased clinical HAV infection in childhood that is why this life threating infection seems more common among adults. Passive immunoprophylaxis also may be one other strategy of HAV prevention in mini epidemic or family outbreak in developing countries. In endemic area human serum immunoglobulin can be used in persons within two weeks after exposure [4]. Immunoglobulin is as effective as vaccination and it should be used in countries that HAV vaccination is not available such as Iran, but it isn't approved for general prevention. In developed countries children receive HAV vaccination at the age of one; however, it may be different in developing countries in which HAV and mother immunity transmitted is endemic and it causes neonatal immunity for long time [5]. It seems that suggestion of vaccination in five year old children in the mentioned article needs to be evaluated more practically.

In conclusion, more studies on seroepidemology of hepatitis A virus are required to evaluate the most appropriate age of HAV vaccination among vhildren in developing countries.
